# What Do We Know about Medication Adherence Interventions in Inflammatory Bowel Disease, Multiple Sclerosis and Rheumatoid Arthritis? A Scoping Review of Randomised Controlled Trials

**DOI:** 10.2147/PPA.S424024

**Published:** 2023-12-13

**Authors:** Kathryn King, Serena McGuinness, Natalie Watson, Christine Norton, Trudie Chalder, Wladyslawa Czuber-Dochan

**Affiliations:** 1 Florence Nightingale Faculty Nursing, Midwifery and Palliative Care, King’s College London, London, UK; 2 Department of Psychological Medicine, Institute of Psychiatry, Psychology and Neuroscience, King’s College London, London, UK

**Keywords:** IBD, MS, RA, treatment, medicine, drug, concordance

## Abstract

**Purpose:**

Between 53% and 75% of people with inflammatory bowel disease, 30%–80% with rheumatoid arthritis, and up to 50% with multiple sclerosis do not take medications as prescribed to maintain remission. This scoping review aimed to identify effective adherence interventions for inflammatory bowel disease, but with few studies found, multiple sclerosis and rheumatoid arthritis were included to learn lessons from other conditions.

**Methods:**

Full and pilot randomised controlled trials testing medication adherence interventions for inflammatory bowel disease, multiple sclerosis, and rheumatoid arthritis conducted between 2012 and 2021 were identified in six electronic databases.

**Results:**

A total of 3024 participants were included from 24 randomised controlled trials: 10 pilot and 14 full studies. Eight investigated inflammatory bowel disease, 12 rheumatoid arthritis, and four multiple sclerosis. Nine studies (37.5%) reported significantly improved medication adherence, all involving tailored, personalised education, advice or counselling by trained health professionals, with five delivered face-to-face and 1:1. Quality of effective interventions was mixed: five rated high quality, two medium and two low quality. Interventions predominantly using technology were likely to be most effective. Secondary tools, such as diaries, calendars and advice sheets, were also efficient in increasing adherence. Only 10 interventions were based on an adherence theory, of which four significantly improved adherence.

**Conclusion:**

Tailored, face-to-face, 1:1 interactions with healthcare professionals were successful at providing personalised adherence support. Accessible, user-friendly technology-based tools supported by calendars and reminders effectively enhanced adherence. Key components of effective interventions should be evaluated and integrated further into clinical practice if viable, whilst being tailored to inflammatory conditions.

## Plain Language Summary

**Introduction**: Inflammatory bowel disease (IBD), multiple sclerosis (MS), and rheumatoid arthritis (RA) are inflammatory diseases where the immune system causes inflammation by mistakenly attacking itself. These lifelong conditions cannot be cured, but can usually be controlled by medication. Medication is most effective when taken as prescribed, called “adherence”. For multiple reasons, between five and eight in 10 people with IBD, RA, and MS do not take medications as advised. This review aimed to identify research that tested ways to help people living with IBD, MS, and RA take medications as prescribed. Identifying and understanding what helps can increase patient well-being and improve quality of life through better controlled inflammatory conditions.

**Methods**: Studies testing medication adherence interventions for IBD, MS, and RA conducted between 2012 and 2021 were identified from six electronic databases.

**Results**: A total of 3024 people with inflammatory conditions took part in 24 studies (eight on IBD, four on MS, and 12 on RA). Nine studies reported significantly improved medication adherence, all involving personalised education, advice or counselling by trained health professionals, with five delivered face-to-face and individually. Interventions predominantly using technology were the most effective. Diaries, calendars, and advice sheets also increased adherence. Ten of the interventions were based on an adherence theory, of which four improved adherence.

**Conclusions**: Personalised adherence support was successful when:

- tailored to patient,
- one-to-one and face-to-face,
- with healthcare professionals,
- involving technology-based tools, and
- supported by calendars/reminders.

Components of effective interventions should be used in clinical practice where possible, made specific to each inflammatory condition.

## Video abstract

**Figure:**
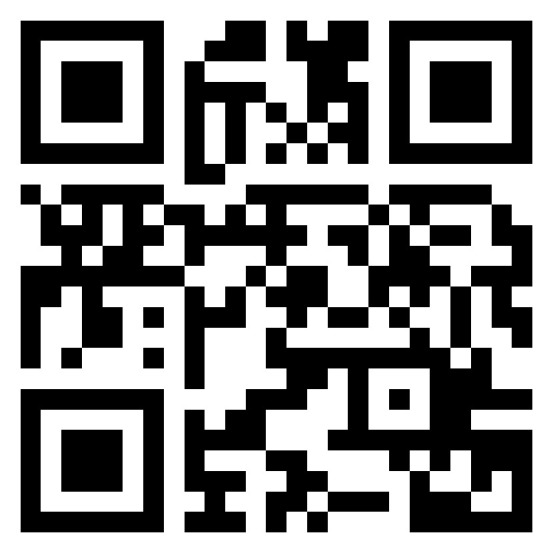

Point your SmartPhone at the code above. If you have a QR code reader the video abstract will appear. Or use:

https://youtu.be/8DY6ZshooKY

## Introduction

Inflammatory diseases comprise a group of chronic conditions where the immune system mistakenly attacks the body’s tissue, causing inflammation.^1^ This can lead to chronic pain, redness, swelling, stiffness, and tissue damage. For the purposes of this review, the inflammatory diseases selected were inflammatory bowel disease (IBD), multiple sclerosis (MS), and rheumatoid arthritis (RA).^2^ All three conditions are incurable, yet have recognised medications to treat, induce, or maintain remission or treat a flare-up, altering the overall disease course for many people.

If treatment is taken as prescribed, for most individuals, outcomes are good, but typically this relies on a high adherence rate. However, like many chronic conditions, non-adherence is problematic in inflammatory diseases, potentially contributing to disease progression and the development of short- and long-term complications. Between 53% and 75% of people with IBD,^3^,^4^ 30%–80% with RA,^5^ and up to 50% with MS^6^,^7^ do not take prescribed medications as advised. Some 15% of individuals with chronic conditions never even redeem their prescriptions.^8^ Therapeutic adherence has also been drastically impacted by the COVID-19 pandemic.^9^ Consequences of non-adherence include increased disease activity and rate of relapse, loss of response to treatment, poor quality of life (QoL), higher disability, morbidity and mortality and additional health expenditure.^3^ Non-adherence is not unique to a specific condition, yet recognising and improving adherence is a primary goal for treatment to keep symptoms quiescent.^10^

In many long-term conditions, when feeling well, an ability to cope “without medication” is often reported.^11^,^12^ This can lead to the adverse effect of poor disease control.^13^ More specifically, due to the fluctuating nature of IBD, for example, being “ill”, has been reported by some people living with IBD as not necessarily a continual state. Certain patients have taken this to mean that medication should only be taken during “illness” or flare-ups.^14^ Being told to take medications regardless of whether an individual feels well or not makes no sense to some, leading to non-adherence. In addition, people living with inflammatory conditions often query the need for pharmacological treatments, voicing concerns about dependence and harmful drug effects.^12^,^14^,^15^

Adherence is influenced by a multitude of interlinked patient-related and healthcare-related factors (eg, symptoms, treatment type, administration route, side effects, medication costs, healthcare beliefs, social circumstances, and culture).^11–13^ These result in multiple barriers to adherence behaviour.^16^ Barriers include perceptual and motivational issues (eg, intentionally missing doses through treatment concerns or wanting to be normal) or practical capabilities (eg, unintentionally forgetting doses). All of these are recognised in the perceptions and practicalities approach (PAPA) for supporting adherence.^17^ The PAPA suggests ways of facilitating adherence. The primary purpose of an adherence intervention is to identify barriers to adherence and target them for individual behaviour change. However, lifestyle change after an inflammatory condition diagnosis can be difficult,^18^ and few adherence interventions acknowledge these barriers.^19^ More specifically for IBD, MS, and RA, despite evidence showing non-adherence is largely associated with psychological factors, including anxiety or depression,^2^,^16^,^20^,^21^ these are often overlooked.^11^ Conversely, interventions designed to reduce anxiety or depression may not target or measure adherence,^10^,^22^ leading to short-term or minimal impact on adherence.^20^,^23–27^ Of those studies that have acknowledged the psychological background to non-adherence, few have explored the role of psychotherapy in improving medication adherence,^28^ yet those that did, found no impact on adherence.^29^

There has been a notable lack of theoretical frameworks addressing behaviour change in adherence research.^3^ Adherence changes have often been transient, minimally impacting longer-term clinical outcomes.^30^,^31^ Most positive results come from complex behaviour change interventions.^30^,^31^ Patient education is also lacking: it is rarely used in adherence promotion^3^ and seldom offered at clinical appointments.^32^ General Practitioners tend not to provide specialist inflammatory condition information and management, which can cause patient–clinician discordance.^3^,^33^ Poor patient understanding of treatment leads to patient frustration, low adherence, and dissatisfaction with care.^34^,^35^

Web-based interventions and telemedicine are favoured by many patients, due to ease of access and reduction in travel.^36^ However, these contemporary interventions have been criticised for lacking tailored, individualised support, resulting in limited information exchange and improvement in adherence.^4^,^18^,^31^,^37^ Interactive interventions with multifaceted education and psychological support have been found to be most efficacious in improving medication adherence.^3^,^38^,^39^ However, effective adherence interventions are rarely used in routine clinical practice.^3^,^4^,^40^

Overall, although there are many interventions designed to increase medication adherence, few have shown long-term effectiveness.^41^ Better management strategies are critical to improve adherence and thereby prevent adverse outcomes, including acute flare-ups and increased disability.^4^,^42^ To achieve this, identifying the most reliable evidence on the effectiveness of adherence intervention components is key. It was anticipated by the authors that there would be a greater number of IBD adherence interventions. However initial searching suggested this was very limited. The search was thus expanded to include the inflammatory conditions of MS and RA.

No previous review has drawn together evidence on the effect of adherence interventions in several conditions. We thus conducted a scoping review in which adherence interventions were evaluated for effectiveness by pilot and efficacy randomised controlled trials (RCTs) in the inflammatory conditions of IBD, MS, and RA. Finally, we identified the intervention techniques used by adherence interventions to feed into the development and evaluation of a new intervention to promote adherence.

## Methods

The aim of this scoping review was to identify and review pilot and efficacy RCTs testing medication adherence interventions initially for IBD, with this subsequently being expanded to MS and RA. All papers written in English published from 2012 with participants ≥18 years of age with a diagnosis of IBD, MS or RA, who were prescribed one or more medications for their condition were included.

### Search Strategy

Six electronic databases (Medline, PubMed, Embase, CINAHL, British Nursing Index, and PsycInfo) were searched systematically in December 2021 to identify published articles from peer-reviewed journals relevant to the review’s aims. Reference lists of included studies were also searched for appropriate papers and duplicates were removed. A combination of terms relating to adherence, the inflammatory conditions (IBD, MS, or RA), and interventions were used to search the databases. (A full list of formatted search terms adapted for each database can be found in Supplementary Table 1).

**Figure 1:**
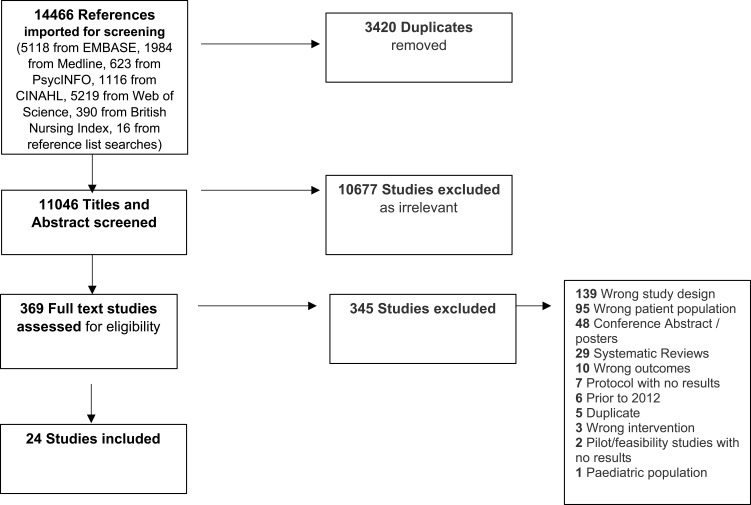
PRISMA flow diagram: selection of studies for scoping review.

**Notes**: PRISMA figure adapted from Moher D, Shamseer L, Clarke M, Ghersi D, Liberati A, Petticrew M, Shekelle P, Stewart LA. Preferred Reporting Items for Systematic Review and Meta-Analysis Protocols (PRISMA-P) 2015 statement. *Syst Rev*. 2015;4(1):1. Creative Commons.^43^

Retrieved studies were exported into EndNote 20 and transferred to Covidence (version 2) reference management software. Three reviewers (KK, SM, NW) screened the titles and abstracts of retrieved papers according to pre-determined inclusion criteria. Two reviewers (SM, NW) were assigned 50% each of the full text papers for data extraction. A third reviewer (KK) performed double data extraction on all included studies, and any disagreements were resolved through discussion with all three reviewers. A flow diagram (Figure 1) reports the selection process and provides reasons for exclusion, as suggested by PRISMA-P guidelines.^43^

### Quality Appraisal

The Critical Appraisal Skills Programme (CASP) tool for RCTs was used to assess the quality of included papers. In line with recommendations,^44^ a CASP scoring system was not used. Instead, a systematic rating system was devised for scoring by the research team. Each study was given a base score of three. One point was subtracted if the study did not use a validated adherence measure and one point was subtracted if the authors did not use intention-to-treat analysis. This resulted in scores of three (high), two (medium), or one (low) quality. No study was excluded based on quality assessment.

### Data Synthesis

Due to the heterogeneity of interventions, it was inappropriate to conduct a meta-analysis of the data. Therefore, studies were synthesised narratively along with descriptive tabulation (**Results,** Table 1).

**Table 1:** Overview of included studies

First author, year; country/continent	Randomised: allocation/arm (all studies are 2 arms, with control arms being TAU unless otherwise stated)	Mean participant age in years (SD or range when SD not stated)	Sex	Population, recruitment setting	Design: primary outcome Completion (ITT or per protocol analysis). Adherence data collection time points	Quality grading of studies (note: studies in bold text had a significantly effective adherence intervention at study completion).
1) Asgari et al^45^ 2021; Iran	Randomised: 200 **Intervention** 100 **Control** 100	**Intervention** 52.4 (±13.6) **Control** 55.0 (±15.4) **Overall** 53.7 *(not stated)*	**Intervention** Male 13 (13%) Female 87 (87%) **Control** Male 11 (11%), Female 89 (89%) **Overall** Male 24 (12%) Female 176 (88%)	RA 2 x Outpatient clinics	RCT; single- blinded (researchers) -Completion: 172 (86%) *(ITT used)* -Baseline, 3 and 6 months	**High**
2) Chapman et al^4^ 2020; United Kingdom	Randomised: 329 **Intervention** 153 **Contro**l 176	**Intervention** 36.0 (27.9–47.1) **Control** 36.8 (28.7–45.1) **Overall** 36.3 *(no mean or SD reportstated, only median and IQR)*	**Intervention** Male 42 (27.4%) Female 111 (72.6%) **Control** Male 49 (27.8%) Female 127 (72.2%) **Overall** Male 91 (27.7%) Female 238 (72.3%)	IBD patient groups, social media, and Outpatient clinics	**Pilot** quasi-RCT *(randomisation process experienced technical error)*; single-blinded (participants) -Completion: 152 (42.6%) *(ITT used)* -Baseline, 1 and 3 months	High
3) Cross et al^46^ 2012; USA	Randomised: 47 **Intervention** 25 **Control** 22	**Intervention** 41.7 (±13.9) **Control** 40.3 (±14.4) **Overall** 41 (±14.0)	**Intervention** Male 10 (40%) Female 15 (60%) **Control** Male 7 (32%), Female 15 (68%) **Overall** Male 17 (36%) Female 30 (64%)	IBD Outpatient clinics	RCT; unblinded (only researchers blinded to group assignment) -Completion: 32 (68.1%) *(ITT and per protocol used, but latter not presented)* -Baseline, 4, 8 and 12 months	High
4) De Jong et al^47^ 2017; Netherlands	Randomised: 909 **Intervention** 465 **Control** 444	**Intervention** 44·0 (±14·1) **Control** 44·1 (±14·2) **Overall** 44.05 *(not stated)*	**Intervention** Male 194 (42%) Female 271 (58%) **Control** Male 180 (41%) Female 264 (59%) **Overall** Male 374 (41.1%) Female 535 (58.9%)	IBD 4 x Outpatient clinics	RCT; unblinded. -Completion: 671 (73.8%) *(ITT used)* -Baseline and 12 months	**High**
5) Del Hoyo et al^48^ 2018; Spain	Randomised: 63 **2x Intervention Arms: Telephone care** 21**Remote Monitoring** 21 **Control** 21	**Telephone care** 40.91 (24–60) **Remote monitoring** 41.32 (19–66) **Control** 39.31 (22–61) **Overall** 40.51 (19–66) *(No mean or SD stated, only median and range)*	**Telephone care** Male 12 (57.1%) Female 9 (42.9%) **Remote monitoring** Male 9 (42.9%) Female 12 (57.1%) **Control** Male 12 (57.1%) Female 9 (42.9%) **Overall** Male 33 (52.4%) Female 30 (47.6%)	IBD Outpatient clinic and inpatient	**Pilot** RCT; unblinded (only researchers during randomisation and statistician blinded) -Completion: No 1° completion data *(ITT used)* -Baseline and 24 weeks	**High**
6) El Miedany et al^49^ 2012; country not stated	Randomised: 147 **Intervention** 74 **Control** 73	**Intervention** 53.2 (±9.6) **Control** 52.8 (±9.5) **Overall** 53 *(not stated)*	**Intervention** Male 21 (28.3%) Female 53 (71.6%) **Control** Male 19 (26.1%) Female 54 (73.9%) **Overall** Male 40 (27.2%) Female 107 (72.8%)	RA Outpatient clinic	RCT; double-blinded -Completion: no 1° completion data *(analysis type not stated)* -Baseline, then every 3 months (18 months duration)	**Low**
7) El Miedany, Gaafary and Palmer^50^ 2012; country not stated	Randomised:111 **Intervention** 55 **Control** 56	**Intervention** 50.50 (±11.2) **Control** 51 (±10.5) **Overall** 50.75 *(not stated)*	**Intervention** Male 14 (25.9%) Female 40 (74.1%) **Control** Male 13 (24%) Female 41 (76%) **Overall** Male 27 (25%) Female 81 (75%)	RA Outpatient clinic	**Pilot** RCT; double-blinded -Completion: no 1° completion data *(analysis type not stated)* -Baseline, then pre-intervention every 3 months (12 months duration)	**Low**
8) Ferguson et al^51^ 2014; United Kingdom	Randomised: 18 **Intervention** 10 **Control** 8	**Intervention** 51 (±14.05) **Control** 46 (±17.04) **Overall** 48.78 (±15.12)	**Intervention** Female 10 (55.6%) **Control** Female 8 (44.4%) **Overall** Female 18 (100%)	RA Outpatient clinic	**Pilot** RCT; unblinded -Completion: 7 (38.9%) *(analysis type not stated; 12 week 1° completion data not used in data analysis. Post-intervention data [6 week] used instead)*. -Baseline, 6 weeks (immediate post-intervention) and 12 weeks	Medium
9) Hebing et al^52^ 2022; Netherlands	Randomised: 206 **Intervention** 104 **Control** 102	**Intervention** 53.5 (±12.1) **Contro**l 51.2 (±13.6) **Overall** 52.4 (*not stated*)	**Intervention** Male 31 (30%) Female 73 (70%) **Control** Male 28 (27%) Female 74 (73%) **Overall** Male 59 (28.6%) Female 147 (71.4%)	RA Outpatient clinic	RCT; unblinded -Completion: no 1° completion data *(ITT used)* -Baseline and every 3 months (12 months duration)	High
10) Keefer at al^53^ 2012; USA	Randomised: 30 **Intervention** 17 *(16 1° Endpoint analysis)* **Control** 13 *(12 1° Endpoint analysis)*	**Intervention** 34.5 (29–39) **Control** 40.8 (31–49) *(No SD stated, only range)* **Overall** 37.7 *(not stated)*	**Overall** Male 10 (30%) Female 18 (70%) *(Not stated/arm, but overall values given).*	IBD Outpatient clinic	**Pilot** RCT; unblinded -Completion: 28 (93.3%) *(analysis type not stated)* -Baseline and 8 weeks	Medium
11) Landtblom et al^36^ 2019; Sweden	Randomised: 93 **Intervention** 46 **Control** 47	**Intervention** 41 (±13.2) **Control** 38 (±10.9) **Overall** 39.5 *(not stated)*	**Intervention** Male 14 (37%), Female 24 (63%) **Control** Male 15 (38%) Female 24 (62%) **Overall** Male 29 (31.2%) Female 48 (51.6%)	MS Outpatient (multicentre)	RCT; single-blinded (participants) -Completion: 53 (57%) *(ITT and per protocol used)* -Baseline, 6 and 12 months	High
12) Linn et al^54^ 2018; Netherlands	Randomised: 160 **Intervention** Part 1: 57 Part 2: 52 **Control** Part 1: 18 Part 2: 33	**Intervention** Part 1: 44.55 (±15.47) Part 2: 40.84 (±14.51) **Control** Part 1 44.11 (±13.86) Part 2: 45.21 (±17.15) **Overall** 43.67 *(not stated)*	**Intervention** Part 1: Male 24 (42.1%) Female 33 (57.9%) Part 2 Male 21 (40.4%) Female 31 (59.6%) **Control** Part 1: Male 6 (33.3%) Female 12 (66.7%) Part 2: Male 17 (51.5%) Female 16 (48.5%) **Overall**: Male 68 (42.5%) Female 92 (57.5%)	IBD 6 x Outpatient clinics	Cluster RCT: participants and research assistants blinded *(not nurses delivering intervention)* -Completion: 98 (61.3%) *(analysis type not stated)* -Baseline, Part 1: 3 weeks Part 2: 6 months	Medium
13) Mary et al^55^ 2019; France	Randomised: 112 **2x Intervention arms**: **Pharmacist Counselling (PC)** 37 **Text Message (TM)** 37 **Control** 38	**Intervention** **PC** 56.3 (±10.6) **TM** 59.1 (±14.4) **Control** 58.2 (±8.8) **Overall** 57.9 (±11.4)	**Intervention** **PC** Male 8 (26.6%) Female 22 (73.3%) **TM** Male 6 (18.8%) Female 26 (81.3%) **Control** Male 7 (20.6%) Female 27 (79.4%) **Overall** Male 21 (21.9%) Female: 75 (78 0.1%)	RA Outpatient clinic	**Pilot** RCT; unblinded -Completion: 96 (85.7%) *(analysis type not stated)* -Baseline, 1 and 6 months	**Medium**
14) Matteson- Kome et al^56^ 2014; USA	Randomised: 6 **Intervention** 4 **Control/Attention control** 2	**Overall** 44.8 (±13) *(Not stated/arm, but overall screened Mean (SD) given).*	**Overall Screened** Male 11 (57.9%) Female 8 (42.1%) *(Not stated/arm, but overall screened values given).*	IBD Outpatient clinic	**Pilot** RCT; single-blinded (participants) -Completion: 5 (83.3%) *(analysis type not stated)* -Baseline, 3 months	Low
15) Nikolaus et al^57^ 2014; Germany	Randomised: 258 *(10 excluded) =* 248 **Intervention** 126 **Control** 122	**Intervention** 46.68 (19.61–88.09) **Control** 44.6 (18.41–81.02) **Overall Median Age** 45.6 *(No mean or SD stated, only median and range)*	**Intervention** Male 68 (54.4%), Female 58 (45.6%) **Control** Male 66 (54.6%), Female 56 (45.4%) **Overall** Male 134 (54%) Female 114 (46%)	IBD Multicentre (18): tertiary referral centres, specialised community hospitals and specialised private practices	RCT; unblinded -Completion: 99 (39.9%) *(ITT used)* -Baseline, week 8 and months 5, 8, 11 and 14	High
16) Rice et al^58^ 2021; USA	Randomised: 85 **Intervention** 43 **Control** 42	**Intervention** 46 (±13) **Control** 44 (±12) **Overall** 44.9 years	**Intervention** Male 11 (26%) Female 32 (74%) **Control** Male 8 (19%) Female 34 (81%) **Overall** Male 19 (22.4%) Female 66 (77.6%)	MS 2 x Outpatient clinics, + virtual recruitment via Zoom	**Pilot** RCT; Unblinded -Completion: 67 (78.8%) *(ITT used)* -Baseline, 90 days	**High**
17) Settle et al^59^ 2016; USA	Randomised: 30 **Intervention** 17 **Control** 13	**Intervention** 51 (±9.2) **Control** 44 (±11.8) **Overall** 47.5 (not stated)	**Intervention** Male 10 (58.8%) Female 7 (41.2%) **Control** Male 5 (38.5%) Female 8 (61.5%) **Overall** Male 15 (50%) Female 15 (50%)	MS Outpatient clinic	**Pilot** RCT; unblinded -Completion: 29 (96.6%) *(analysis type not stated)* -Baseline, 3 and 6 months	Medium
18) Song et al^60^ 2020; China	Randomised: 92 **Intervention** 46 **Control** 46	**Intervention** 57.05 (±11.31) **Control** 53.22 (±10.04) **Overall** 55.26 (±10.84)	**Intervention** Male 11 (26.8%) Female 30 (73.2%) **Control** Male 11 (30.6%) Female 25 (69.4%) **Overall** Male 22 (28.6%) Female 55 (71.4%)	RA Rheumatology Outpatient department, tertiary hospital	RCT; unblinded -Completion: 77 (83.7%) *(analysis type not stated)* -Baseline, 12 and 24 weeks	**Medium**
19) Taibanguay et al^61^ 2019; Thailand	Total:120 **Multicomponent intervention** 60 **Single intervention/control** 60	**Multiple interventions** 55.82 (±11.25) **Single intervention/control** 57.20 (±12.24) **Overall** 56.5 *(not stated)*	**Multiple interventions** Male 9 (15%) Female 51 (85%) **Single Intervention/control** Male 10 (16.9%) Female 49 (83.1%) **Overall** Male 19 (16%) Female 100 (84%)	RA Rheumatology Outpatient clinic	RCT; single-blinded (Assessed by blinded rheumatologist for 2° outcomes) -Completion: 119 (99.2%) *(ITT used)* -Baseline, 12 weeks	High
20) Tan et al^62^ 2021; Singapore	Randomised: 132 **Intervention** 66 **Control** 66	**Intervention** 49.18 (± 12.03) **Control** 50.68 (±12.45) **Overall** 49.9 *(not stated)*	**Intervention** Male 7 (11.29%) Female 55 (88.71%) **Control** Male 11 (17.19%) Female 53 (82.81%) **Overall** Male 18 (14.24%) Female 108 (85.76%)	RA Rheumatology Outpatient clinic	RCT; single-blinded (rheumatology nurse only, who conducted clinical assessments) -Completion: 119 (90.1%) *(ITT used)* -Baseline, 1, 3 and 6 months	High
21) Turner et al^63^ 2014; USA	Randomised: 19 **Intervention** 12 **Control** 7	**Intervention** 50.75 (±8.18) **Control** 55.29 (±4.92) **Overall** 53.02 (*not stated)*	**Intervention** Male 10 (83.3%) Female 2 (16.7%) **Control** Male 6 (85.71%) Female 1 (14.29%) **Overall** Male 16 (84.2%) Female 3 (15.8%)	MS Outpatient clinic	**Pilot** RCT; single-blinded (researcher only) -Completion: 19 (100%) *(ITT used)* -Baseline, 1, 3 and 6 months	**Medium**
22) Unk et al^64^ 2014; USA	Randomised: 108 **Intervention** 54 **Control** 54	**Intervention** 50.1 (±12.9) **Control** 50.5 (±11.3) **Overall** 50.3 (±12.1)	**Intervention** Male 8 (14.8%) Female 46 (85.2%) **Control** Male 14 (24.9%) Female 40 (74.1%) **Overall** Male 22 (20%) Female 86 (80%)	RA Outpatient clinic	RCT; unblinded -Completion: 98 (91%) *(Analysis type not stated)* -Baseline, 1 month	Medium
23) van Heuckelum et al^65^ 2021; Netherlands	Randomised: 93 **Intervention** 47 **Control** 46	**Intervention** 58.1 (±13.6) **Control** 59.9 (±13.9) **Overall** 59 (not stated)	**Intervention** Male 14 (29.8%) Female 33 (70.2%) **Control** Male 18 (39.1%) Female 28 (60.9%) **Overall** Male 32 (34.4%) Female 61 (65.6%)	RA 2 x Community- based centres	Open-label RCT; Unblinded -Completion: 51 (54.8%) *(ITT used)* -Baseline, 12 months	High
24) Zwikker et al^66^ 2014; Netherlands	Randomised: 123 **Intervention** 63 **Control** 60	**Intervention** 60.4 (±12.1) **Control** 59.3 (±11.3) **Overall** 59 (not stated)	**Intervention** Male 21 (33.3%) Female 42 (66.7%) **Control** Male 17 (28.3%) Female 43 (71.7%) **Overall** Male 38 (30.8%) Female 85 (69.2%)	RA Single centre: specialist rheumatology, rehabilitation, and orthopaedic clinics	RCT; single-blinded (researcher only) -Completion: 106 (86.2%%) *(ITT used)* -Baseline, 1 week, 6 months and 12 months (post-second group session)	High

**Abbreviations**: IBD, inflammatory bowel disease; IQR, interquartile range; ITT, intention-to-treat; MS, multiple sclerosis; PC, pharmacist counselling; RA, rheumatoid arthritis; RCT, randomised controlled trial; TAU, treatment as usual; TM, text message; USA, United States of America.

## Results

A total of 14,466 papers were identified from six databases and from searching reference lists of included studies. After screening titles and abstracts, 369 papers remained for full text eligibility screening. A total of 24 papers were included in the scoping review and underwent data extraction (Table 1).

### Demographics

Across the 24 studies, eight (33.3%) investigated IBD, four (16.7%) MS and 12 (50%) RA. Eleven studies were conducted in Europe (45.8%), including two in the UK (8%), with seven in the US (29%), four (16.6%) in Asia (Iran, China, Thailand and Singapore), and two studies did not report study country/continent. In total, 3024 participants were randomised, ranging from 18 to 909 per study. Participant ages ranged from 18 to 81 years, and all studies included both male and female participants, except one, which had 100% female participants.^51^

In terms of design, 10 (41.7%) studies were pilot RCTs (and thus may not be reasonably expected to reach statistical significance), with the remainder being efficacy RCTs. Studies largely had two arms and were delivered at a single centre. Two studies reported double-blinding, four did not report blinding, nine studies were unblinded, and the remaining nine used variants of single-blinding. Intervention length spanned from 15 minutes to 12 weeks, with follow-up duration ranging from six weeks to 18 months from randomisation. Table 1 gives further details of the included studies and Table 2 shows specific information regarding the interventions.

**Table 2:** Description of interventions

Author/s, Year, Inflammatory Disease	Study Objective	Theoretical background of intervention components	Intervention Details and Completion	Measure of Adherence	Key Findings
Asgari et al^45^ 2021, RA	To design and evaluate a theory-based intervention to improve medication adherence among RA patients.	Health action process approach	Intervention: 3×40 minute face-to-face sessions, 1 week apart using behaviour change techniques delivered by therapist. Control: treatment as usual (TAU).Intervention completion:**Intervention** 100 (100%)**Control** 100 (100%)	*MARS*	Intervention group reported significantly greater improvements in *MARS* scores at both 3 and 6 months (*P*<0.001) compared to control arm.Indirect mediation effects of theory-based self-regulation factors (medication beliefs, intention, coping planning, self-monitoring, and behavioural automaticity) were largely significant mediators of an intervention effect on medication adherence scores (*P*<0.001).
Chapman et al^4^ 2020, IBD	To pilot the development and evaluation of a *PAPA-*based intervention focusing on:a] capacity to change perceptual and practical barriers to adherence;b] online feasibility;c] patient acceptability.	*PAPA*	Intervention group: tailored, personalised online intervention to address beliefs about IBD, medication and treatment and provide advice. Control group: *TAU*.Intervention completion:Not stated	*MARS; Adherence VAS*	No significant difference between intervention group and controls on the *MARS* adherence measure.However, patients in intervention group showed higher *VAS* adherence than controls.-For azathioprine, adherence was higher at 1 and 3 months on the *VAS* (*P*=0.03).Intervention group was more satisfied with information about IBD medication at both follow-up points (*P*<0.05) and about action and medication usage at 1 month (*P*<0.05), as opposed to controls.However, all participants reported only one practical barrier to adherence on recruitment and median baseline *VAS* adherence score was 100% (as it was for both follow-ups, groups and medications), meaning no capacity for adherence to improve.
Cross et al^46^ 2012, IBD	To evaluate effect of a Home automated telehealth system (*HAT)* for ulcerative colitis on disease activity, *QoL* and adherence compared to best available care in a *RCT*.	None stated	Intervention: HAT system involving weekly questions on recent symptoms, well-being, side effects and adherence. Prompts sent to intervention participants for guidance. Customised action plans created and disease specific education. Educational curriculum (provided by Crohn’s and Colitis Foundation of America) delivered after each session. Nurse contactable any time. Message alerts sent to nurse if data incomplete. Control: *TAU*; routine follow-up, received written action plans and educational fact sheets. Intervention completion:**Intervention** 22 (88%) **Control** 19 (86.4%)	*MMAS*	No significant difference between intervention and control group.However, completer analysis revealed higher adherence rates in intervention group compared to *ITT* analysis.
De Jong et al^47^ 2017, IBD	To compare a telemedicine system vs *TAU* Outpatient care for patients with IBD.	None stated	Intervention: Web-based telemedicine intervention accessible via smartphone or tablet, available for 12 months. Outpatient and e-learning modules, personal care plan, questions and measures. Participant monitoring of disease activity facilitated through red flags: alert sent to healthcare provider, leading to 1:1 contact with participant. Messaging system involved healthcare provider. Control: *TAU;* routine follow-up with opportunity to schedule extra visit if symptoms relapsed. Intervention completion:**Intervention** 438 (94%) **Control** 443 (99.8%)	*MMAS*	Adherence to medication at 12 months was significantly higher in telemedicine group than in control group (*P*<0.001).Perceived knowledge of medication was scored highly by both participant groups, with no significant differences between groups.
Del Hoyo et al^48^ 2018, IBD	To pilot evaluating impact of remote monitoring using a web system compared to treatment as usual and telephone care on health outcomes and healthcare in patients with complex IBD.	None stated	24 week study: Interventions: Remote monitoring web-based platform with advice, reminders, educational material (web and paper-based) and information on prevention. Participants invited to input information to system, accessible to clinical staff, who could feed back accordingly.Telephone care via nurse with interventions based around telephone interviews. Patients provided with paper-based educational material. Control: *TAU* in IBD unit, paper-based educational information provided and clinical activity logged in paper diary.Intervention completion:I) **Remote monitoring** 18 (85.7%)II) **Telephone care** 20 (95.2%)III) **Control** 19 (90.5%)	*MMAS*	Medication adherence improved significantly (*P*≤0.05) in 3 arms at 24 weeks:I) **Remote monitoring**: 57.1% →85.7%II) **Telephone care**: 33.3% →71.4%III) **Control**: 66.7%→ 81%Reduction in MMAS was more significant in remote monitoring than in control group (*P* ≤0.05)All completers adhered to treatment in remote-monitoring arm (MMAS score=0), but not in telephone care or control group.
El Miedany et al^49^ 2012, RA	To assess integration of patient reported outcome measures (*PROMs)* and patient education, using a joint-fitness programme, and effectiveness of this combined approach on disease activity and adherence to therapy.	*CBT* theory	1:1 clinical examination and assessments, with 3 month intervals of data recording.@Month 6 of treatment, participants randomly allocated to: Intervention: given face-to-face 1:1 sessions with rheumatologist to discuss problems and set health-related goals for 12 months. Outcome measures discussed with each patient. Fitness programme followed, then post-treatment questionnaire. Control: *TAU;* continued treatment and management based on *PROMS* and clinical assessment. Intervention completion:Not stated	Study specific medication taking behaviour parameters	89% of intervention group were reportedly adherent to medication compared to 64.4% of control group. Difference was significant (*P*<0.01) at 18 months.Significant reduction in intervention group (40.5%) in number of clinic procedures and visits for flare-ups requiring early assessment, as opposed to controls (73.9%) at 18 months.Based upon post-treatment questionnaire, intervention participants were significantly less likely to cease medication due to intolerance, more able to cope with *ADLs*, with fewer future concerns.
El Miedany, Gaafary and Palmer^50^ 2012, RA	To pilot evaluating the feasibility of using visual feedback in patients with early inflammatory arthritis, its effect on adherence to therapy and disease activity and to assess how ubiquitous computing technology can improve therapy compliance and adherence.	None stated	1:1 Clinical examination and assessments, with 3 month intervals of data recording.@Month 6 of treatment, patients randomly allocated to: Intervention: visual feedback (visualisation of charts showing progression of disease activity parameters) was added to management protocol within clinical examination for 6 months. Control: *TAU;* continued standard management protocol for 6 months. Outcome measures discussed 1:1 with each patient.Intervention completion:Not stated(Note: author contacted multiple times for further information but no response).	Blood checks to monitor medication levels	At 12 months, patients in intervention group (92.7%) were significantly (*P*<0.01) more adherent to medication than those in control group (69.6%).Intervention group were less likely to stop medication due to intolerance, and more able to cope with *ADLs,* with fewer future concerns (*P*<0.01)Medication adherence was significantly correlated (*P*<0.01) with changes in all measured disease parameters; highest correlation with *QoL* (0.460) followed by patient global assessment (0.433), functional disability (0.340), disease activity (0.324) and pain (0.313).
Ferguson et al^51^ 2014, RA	To pilot the adaptation of a psychological intervention to improve medication adherence for patients with RA and evaluate their intervention experience.	*CBT*/*MI*	2-arm pilot study: Intervention: up to 6 weekly, manualised 50 minute 1:1 sessions of *CBT/MI* with a psychologist, focusing on: i) practical/perceptual factors impacting upon adherence; ii) ambivalence towards complex and long-term medications regimen; iii) pros/cons of alternative courses of action and medication benefits; iv) challenging/modifying unhelpful treatment and illness beliefs. Control: *TAU.* Intervention completion: Not stated	*MARS and MMAS*	No significant effect between intervention group versus controls for 1° or 2° outcomes.Intervention group demonstrated significant within-group differences for mean scores from baseline to immediately post-intervention; *MARS* (*P*=0.022) and *MMAS* (P=0.049).Qualitative feedback (not explicitly reported) suggested intervention helped proactive medication management.(Note: Minimal data presented post 6 weeks intervention due to low response rate. Also, unclear reporting of time points due to multiple anomalies).
Hebing et al^52^ 2022, RA	To assess effectiveness of electronic monitoring feedback (*EMF)* on medication adherence in patients with RA starting with or switching to new biological disease modifying antirheumatic drugs (*bDMARDs).*	None stated	Participants assigned to group and monitored for 12 months. Intervention: given needle-disposal container equipped with medication event monitoring system (*MEMS). MEMS* scores calculated every 3 months for 12 months, with 1:1 *MI* feedback given by pharmacists/technicians. Non-adherence counselling given following a semistructured model. Control: *TAU.* Intervention completion: Not stated	*MEMS*(used to calculate medication possession ratio; *MPR*)	No significant difference between intervention group and controls.Adverse effects of *bDMARDs* reported by 52% of participants. 42% ceased or switched due to side effects, loss of effect or other reasons.
Keefer at al^52^ 2012, IBD	To pilot determining feasibility/acceptability and estimate effects of a program of project management on *CD* outcomes to a non-traditional group of *CD* patients.To optimise management of *CD* by addressing health behaviours that undermine medical therapies, increase risk of disease flare and hinder *QoL*.	Health behaviour change and social learning theory	Intervention: “Project management” with 6 weekly, 60 minute individualised sessions with a health psychologist, following a personalised self-management protocol “fostering ritualistic and habitual health behaviours.” +Formal instruction in relaxation training and nutritional consultation with dietician. Control: *TAU.* Intervention completion:Not stated	*MMAS*	No significant difference in either group for medication adherence.Significant differences found between groups on self-reported *QoL*, perceived stress, self-efficacy andmedication adherence effect (*P*=0.02).
Landtblom et al^36^ 2019, MS	To investigate impact of a (tele)medicine patient support programme (*MSP)* concerning health-related QoL and adherence in patients with relapse–remitting MS, being administered Rebif, using the RebiSmart device.	None stated	Intervention: 12 month patient support programme. Participants received phone calls, text messages and emails from MS nurse support coach covering a range of topics, including treatment adherence. Access to web-based journal provided to track progress and get advice on RebiSmart device. Control: *TAU* + technical support given for Rebismart device usage, but no access to programme. Intervention completion:**Intervention**: 38 (82.6%) **Control**: 39 (83%)	RebiSmart Device	No significant difference between intervention and control group.
Linn et al^53^ 2018, IBD	To test synergistic effects of an evidence-based Tailored Multimedia Intervention (*TMI)* with technology (online preparatory assessment and text messaging) as an add on to a tailored counselling session.	*Elaboration likelihood model*	Intervention:Two parts: Part 1: Participants received a 1:1 30 minute nurse counselling session about newly prescribed medication.Sites randomised.Part 2: Experimental sites: received *TMI*.New patients completed online preparation tool identifying adherence barriers and attended 1:1 nurse consultation (nurses trained in communication skills).Participants perceiving adherence barriers at baseline or 3 weeks were sent weekly text messages for 6 months, designed to change barriers in a direction more consistent with higher adherence. Control sites: *TAU* (standard education). Intervention completion: **Intervention: Part 1** 36 (63.2%); **Part 2** 28 (53.8%); **Control: Part 1** 12 (66.7%); **Part 2** 22 (66.7%)	*MARS*	No significant difference between intervention and control group.However intervention nurses’ affective communication (making participant feel respected, known and understood) was rated more highly three weeks after intervention, as opposed to control group.
Mary et al^54^ 2019, RA	To pilot the impact of weekly text messages on adherence in patients taking methotrexate *(MTX)* for RA.	None stated	Three groups (6 month intervention): *Intervention Pharmacist Counselling (PC): TAU* standard 1:1 consultation + 15 mins 1:1 counselling session with pharmacist + standardised advice sheet. *Intervention Text Message (TM): TAU* standard 1:1 consultation +standardised weekly *TM* reminders. Control: *TAU* standard 1:1 consultation. Intervention completion:**Intervention PC** 30 (81.1%)**Intervention TM** 32 (86.5%)**Control** 34 (89.5%)(Note: author contacted multiple times for further information but no response).	1° measures: *Compliance Questionnaire Rheumatology(CQR-19*);2° measures:Girerd questionnaire and *MPR* based on *MTX* prescription renewals and number of *MTX* units in patient’s possession/visit.	1° measure: At endpoint, participants in *TM* group showed significantly higher levels of adherence than control group (*P*=0.019). Difference not seen for *PC* group.2° measure: Proportion of adherent participants was 56% in control group, 53% in PC group and 78% in TM group. Significant between-group differences (*P*<0.025) defined by combination of 1° and 2° measures.Intergroup differences were not significant for Girerd score alone.In addition, patient satisfaction was significantly higher for intervention groups than control group (*P*<0.01).
Matteson-Kome et al^55^ 2014, IBD	To pilot evaluating feasibility, intervention mechanism, and potential effectiveness of a 3 month continuous self-improvement intervention to enhance medication adherence in adult non-adherent IBD patients.	Systems theory	*MEMS* caps and *MEMS* diaries given to all participants. Intervention: participants received personal-system theory PowerPoint presentation in IBD clinic, followed by 1 x face-to-face 20 to 45 minute intervention. *MEMS* data analysed by principal investigator and participant together for non-adherence patterns. Behaviour change and potential changes identified over 3 months. Control: participants received 1 x face-to-face educational 39 to 40 minute session by principal investigator with electronic slide presentation and IBD educational handout. Intervention completion:Overall, 5/6 randomised participants completed. (Individual arms not stated).	*MEMS* caps and*MEMS* diaries	No significant difference between intervention and control group.(Note: small sample, control group not well matched, short dose and duration of intervention).
Nikolaus et al^56^ 2014, IBD	To compare durable adherence to mesalamine treatment between patients undergoing education vs treatment as usual.	None stated	Intervention: 2 hour education session delivered between day 0 and week 4 by a nurse/research physician with prior training, using standardised slides. Education covered aetiology of ulcerative colitis, disease course, complications, therapy regimen (necessity and benefits of mesalamine) and strategies to prevent acute relapses. A group session followed, where participants could ask questions and were given contact methods for individual queries. Control: *TAU*, then offered programme following trial completion. Intervention completion:Not stated (note: author contacted multiple times for further information but no response).	*MMAS*;short-term adherence:urine levels of 5-ASA and metabolite(N-acetyl-5-ASA)	No significant difference between intervention and control group.
Rice et al^57^ 2021, MS	To pilot test the impact of electronic pill bottles with audio-visual reminders on oral disease modifying therapy *(DMT)* adherence in people with MS.	None stated	90 day intervention: all participants given baseline tutorial with choice as to when to receive pill taking reminders. Intervention: access to remote smartphone application (“Pillsy Bottle”) with feedback and electronic pill bottle with medication reminders. Control: TAU + electronic pill bottle cap only.Intervention completion:**Intervention** 42 (97.7%)**Control** 39 (92.9%)	Electronic pill bottle(no. of pills taken within ±3 hours/total pills consumed by participant during study; converted to %).	Participants in control group had significantly more pills taken late or missed (***P*=0.033**).Participants who took fewer than 3 pills/day, had a significantly higher optimal average adherence than those taking more than 3 pills/day (***P*=0.04**).
Settle et al^58^ 2016, MS	To pilot employing a web-based system to monitor and potentially modify MS medication adherence.	None stated	Intervention: 6 month internet-based module supporting patient self-management, patient-provider communication and patient education, targeting needs of MS patients and providers. Text/email reminders set-up to administer intramuscular MS meds (IFNβ_1a_) on chosen day. Weekly probe sent, asking how many days that week vitamin D taken. Control: *TAU*.Intervention completion:**Intervention** 15 (88.2%)**Control** 10 (76.9%)	*MMAS-8*, *MS-HAT* alerts, calendar reports, syringe counts, pharmacy refills, blood serum levels and self-reported adherence.	No significant difference between intervention and control group.
Song et al^59^ 2020, RA	To explore effects of a tailored educational intervention via telephone on medication adherence and disease activity in discharged patients with RA.	Health Belief Model, evidence-based guidelines, expert advice and literature	Intervention: 4 x tailored telehealth educational sessions, lasting 20–40 minutes, via telephone across 12 weeks (weeks 2, 4, 8, 12 post-discharge). Control: *TAU* consisting of discharge instructions (medication guidance and basic health advice for RA patients).Intervention completion:**Intervention** (93.5%) **Control** 39 (85%)	*Compliance Questionnaire Rheumatology - 19* (*CQR-19; Chinese version*)	Intervention group had significantly higher medication adherence compared with control group at 12th **(P=0.014)** and 24th week **(P=0.042)**.(Note: Use of a randomised post-test design did not provide any baseline data regarding initial adherence levels).
Taibanguay et al^60^ 2019, RA	To assess the influence of different modes of patient education on medication adherence in patients with RA.	None stated	Intervention: 30 minute directed counselling and a disease-information pamphlet. Control: received disease-information pamphlet only.Intervention completion:**Intervention** 60 (100%)**Control** 60 (100%)	Pill count and translated medication taking behaviour questionnaire (set of standardised self-reporting measures).	No significant difference between intervention and control group.
Tan et al^61^ 2021, RA	To evaluate effect of a musculoskeletal ultrasound programme *(MUSP)* in *RA*, using real-time ultrasonography with rheumatologist advice on improving *bDMARD* adherence.To evaluate *MUSP*’s patient feasibility and acceptability.	None stated	Intervention: MUSP session (mean time 9.2 minutes) with a rheumatologist using standardised messages, to explain disease progression and drug role in prevention of RA, whilst improving understanding. Control: *TAU* (no *MUSP*).Intervention completion:**Intervention** 62 (93.9%)**Control** 64 (97%)	*MMAS-8;* pill count	No significant difference between intervention and control group at month 3 and month 6.However, proportion of participants with low adherence in intervention group was significantly lower than control group **(P=0.019)** at month 1.
Turner et al^62^ 2014, MS	To pilot the evaluative impact of brief telephone-based counselling using principles of *MI* and telehealth home monitoring on medication adherence.	Transtheoretical model/*MI*	Participants enrolled in a longitudinal cohort study who endorsed non-adherence were invited to take part in pilot. Intervention: 3 x telephone counselling sessions (45–75 minutes each) within 6 months. Home telehealth monitoring set up between session 2 and 3 to deliver tailored text messages. Control: *TAU*. Offered telephone counselling and monitoring after completion of final follow-up.Intervention completion:**Intervention** 19 (100%)**Control** 19 (100%)	Adapted self-report questionnaire (calculating missed medication doses).	At 6 months, participants in intervention group reported higher levels of adherence than those in control **(P<0.05)**.(Note: differences between intervention and control were large, consistent and increased over time but partly limited by baseline differences).
Unk et al^63^ 2014, RA	To compareadherence impact of a multimedia presentation vs standard educational literature.	Cognitive theory of multimedia learning process	Intervention: multimedia presentation — 15 minute programme containing 5 topics (cause of RA, impact on body, treatments, healthy self-care and additional resources). Information copies given to take away for review. Control: received literature about RA from a national RA organisation, containing similar information.Intervention completion:Not stated	*MAQ*	No significant difference between intervention and control group.(Note: limitations in use of and understanding of MAQ by participants, plus increased awareness of meaning to MAQ were acknowledged as potentially an impact upon outcomes).
van Heuckelum et al^64^ 2021, RA	To study effectiveness of electronic monitoring feedback *(EMF)* in patients with early RA to improve medication adherence and clinical outcomes vs treatment as usual.2° objective: examine intervention effectiveness on patients’ disease activity, health status, beliefs about medicines and time to first anti-TNFα (tumor necrosis factor) prescription.	None stated	Both groups included 3 monthly follow-up appointment with rheumatologists up to 12 months. Intervention: all medication dispensed in Electronic Drug Monitors providing feedback. *EMF* given by MI-trained pharmacists prior to regular consultations. Control: TAU (consultation with pharmacy consultant prior to visiting rheumatologist without electronic monitors and EMF).Intervention completion:**Intervention** 27 (42.5%)**Control** 29 (63%)	*CQR19;* *MMAS-8*	No significant difference between intervention and control group.
Zwikker et al^66^ 2014, RA	To assess effect of a group-based intervention on balance between necessity beliefs and concerns about medication and on medication non-adherence in patients with RA.	None stated (note: systematic development of intervention acknowledges the “Intervention Mapping” framework)^67^	Non-adherent patients (taking ≤80% of prescribed medication according to *CQR*) were invited to partake. Intervention: 2 x motivational interviewing-guided group sessions led by pharmacist, designed to improve participants’ balance between necessity beliefs and concern beliefs about medication and resolve medication taking practical barriers. Sessions one week apart with 5–7 RA attendees. Control: received brochures regarding medications and requested to thoroughly read brochures.Intervention completion:**Intervention** 57 (90.5%)**Control** 60 (100%)	*CQR-19;* *MARS* + pharmacy refill data to calculate *MPR.*	No significant difference between intervention and control group.At 12 months, intervention participants had less strong necessity beliefs about medication than control participants.

**Abbreviations**: ADLs, activities of daily living; bDMARD, biological disease-modifying antirheumatic drug; CBT, cognitive behavioural therapy; CD, Crohn’s disease; CQR-19, Compliance Questionnaire Rheumatology; DMT, disease modifying therapy; EMF, electronic monitoring feedback; HAT, home automated telehealth system; IBD, inflammatory bowel disease; MARS, Medication Adherence Report Scale; ITT, intention-to-treat; MAQ, Medication Adherence Questionnaire; MEMS, Medication Event Monitoring System; MI, motivational interviewing; MMAS, Morisky Medication Adherence Scale; MPR, medication possession ratio; MS, multiple sclerosis; MSP, (tele)medicine patient support programme; MTX, methotrexate; MUSP, musculoskeletal ultrasound programme; PROMs, patient reported outcome measures; QoL, quality of life; RA, rheumatoid arthritis; RCT, randomised controlled trial; TAU, treatment as usual; TMI, Tailored Multimedia Intervention; VAS, visual analogue scale.

### Adherence Improvements

Four full RCTs showed a significant improvement in medication adherence,^45^,^47^,^49^,^59^ as did five pilot RCTs^48^,^50^,^54^,^57^,^62^ (Table 2). These full RCT interventions ranged from three 40 minute sessions over 3 weeks to a 12 month period, with 6−18-month follow-up. For the five pilot studies, interventions lasted from 90 days to 12 months, with follow-up of 3–12 months. One additional pilot study found significant outcomes measured by one adherence measure but not another.^4^ Intervention access and follow-up lasted 3 months.

There were also two (16.7%) pilot RCTs^51^,^58^ and two full RCTs^60^,^61^ with statistically significant adherence improvements shown during the course of follow-up, but not at the final post-intervention time point. Interventions for these full RCTs ran from an average of 9.2 minutes to 30 minutes, with follow-up from 12 weeks to 6 months. These pilot interventions ranged from 6 weeks to 6 months, with follow-up periods of 12 weeks to 6 months.^51^,^58^ However, one pilot study did not specify their primary outcome time point.^58^

All nine significantly effective interventions involved face-to-face or remote interaction with a healthcare professional throughout the intervention (separately from data collection staff). Either nurses,^47^,^48^,^59^ gastroenterologists,^47^ a therapist,^62^ pharmacist,^54^ researchers^45^,^57^ and rheumatologists^49^,^52^ were trained in an approach for intervention delivery. In one study, beneficial effects of involving nurses with knowledge of RA combined with delivery of patient-centred education was demonstrated.^59^ In another study using a web-based platform, continuous communication between patients and health providers was facilitated via electronic messaging, resulting in significant improvement in medication adherence.^48^ Adherence reached 100% in participants completing the web-based platform. This was attributed by the authors to continual adaptation of care in relation to the participant’s disease activity and optimal communication.

Effective interventions mostly incorporated a form of tailoring with educational support for participants, where they had opportunities to ask questions about their needs. Interactive education, counselling, goal setting and a joint fitness programme centred around RA resulted in significantly improved medication adherence.^49^ Monitoring with tailored support for participants with IBD led to positive, significant adherence effects, reduced social impairment and daily activity interference.^45^,^51^ Patient satisfaction was high and health-related QoL improved. Similarly, success of a tailored health behaviour change intervention to fit the needs of participants living with RA, including health literacy was also reported.^45^ Personalised tailoring was applied by another study,^59^ enabling intervention participants to have their individual needs as the central focus. Primary outcome measures indicated a significant increase in adherence rates for intervention participants, both at 12 and 24 weeks.

**Figure 2:**
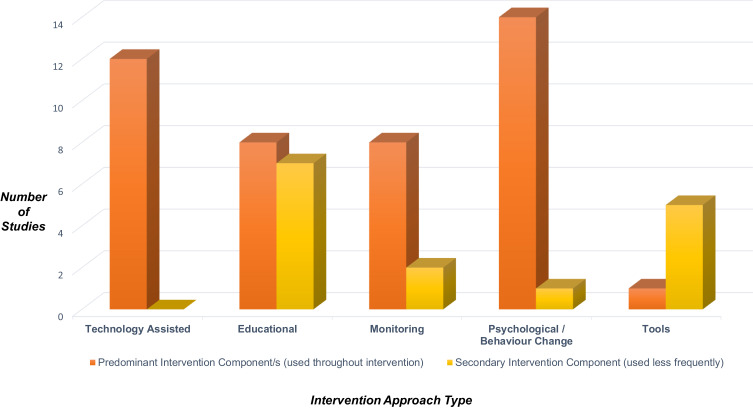
Categories of interventions.

**Figure 3:**
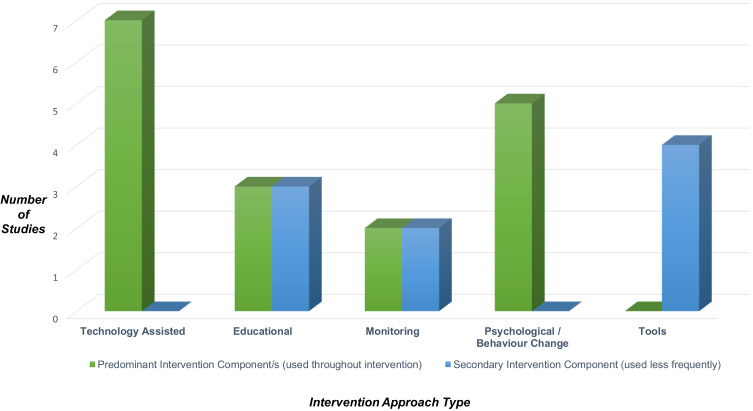
Categories of significantly effective interventions.

Over half (five of nine) of successful interventions were delivered face-to-face and 1:1.^45^,^49^,^50^,^59^,^62^ A range of approaches were used, including principles of cognitive behavioural therapy (CBT),^49^ motivational interviewing (MI)^59^ and visual feedback^49^,^52^ (see Table 2 and Figures 2 and 3; outlining intervention approaches used). Of the studies with statistically significant changes in adherence, all those involving a face-to-face intervention were graded highly in terms of quality,^45^,^47^,^48^,^57^ with one exception graded as medium.^54^ Interventions without a face-to-face approach were rated medium^59^,^62^ or low quality.^49^,^52^

### Adherence Measurement

The most common method of adherence measurement was via a self-report questionnaire. The Morisky Medication Adherence Scale (MMAS)^68^ was used in 10 studies, followed by the Medication Adherence Report Scale (MARS)^69^ in five studies. One intervention collated data via study-specific medication taking behaviours (for example, whether medication was stopped due to intolerance) to measure adherence.^49^ Four studies included pill and/or syringe counts to assess adherence,^58^,^60^,^61^,^64^ whereas only three used electronic medication monitoring,^54^,^57^,^65^ despite the latter approach being considered the “gold standard” of adherence monitoring.^71–76^ Objective physiological measures (blood and urine tests), calendars and diaries were less commonly used, and almost half the studies used a combination of methods to measure adherence.^4^,^51^,^54–61^,^64^,^68^

### Ineffective Interventions

Eight studies showed no significant effect on medication adherence,^36^,^46^,^52^,^53^,^55^,^56^,^63^,^65^ but two of these were pilot studies only.^52^,^55^ These varied across length of intervention from 15 minutes^63^ to 12 months^36^,^46^,^64–66^ and follow-up from 1 month^63^ to 14 months.^56^ Sample size and attrition rate, use of theory and approach were also variable amongst these interventions. Within these eight, the two pilot studies^52^,^53^ and two of the full RCTs utilised behaviour change and psychological methods,^46^,^65^ and four applied education^36^,^46^,^56^,^63^ as intervention components.

An additional two full RCT interventions showed no significant difference compared to controls but also had detrimental effects on adherence at completion or on adherence-related beliefs.^64^,^66^ However both studies were graded high quality, interventions were not theoretically-based and involved pharmacists delivering motivational interviewing. Each study was 12 months duration, utilising the Compliance Questionnaire Rheumatology measure of adherence.^76^

### Inflammatory Condition Type

The eight IBD interventions showed a variable success rate. The two effective IBD interventions utilised web-based interventions, one full RCT offering education modules and “red flag” monitoring of disease activity, with a web-based platform compared with telephone education.^47^ The effective pilot study used text message reminders with direct tailored feedback.^48^

For the four MS interventions, home-based coaching support with nurses, including a web-based journal to track participants’ progress with the Rebismart device led to adherence improvements in the intervention group.^36^ However, these were non-significant between groups. Similarly, an internet-based pilot programme delivering personalised text or email adherence reminders showed a moderate effect on adherence for only participants living alone and not in the main analysis.^58^ Pilot studies involving both an electronic pill bottle cap with audio-visual medication reminders^57^ and motivational interviewing–based telephone counselling^62^ led to significant adherence improvements for participants with MS compared with controls.

For the 12 RA interventions, five (41.6%) studies were effective,^45^,^49^,^50^,^54^,^59^ including two pilot interventions.^50^,^54^ Of the five studies that showed significant differences between groups, three used psychological behaviour change approaches.^45^,^49^,^59^ A mixture of educational and technological techniques were also successful through telephone-education sessions with trained nurses,^59^ participants viewing their disease progression on the clinical computer system^50^ or receiving weekly text message reminders to take RA medication.^54^

### Multicomponent Interventions

All nine significantly effective interventions^45^,^47–50^,^54^,^57^,^59^,^62^ utilised two or more components within their intervention, often with a predominant component and then a secondary one (Figures 2 and 3. See Supplementary Table 2 for more detail). Two interventions incorporated a combination of technology, education, monitoring and web-based platforms that registered disease activity and adherence.^47^,^48^ Progression of IBD activity, medication use, body weight, vital signs and testing schedules were logged,^48^ all of which were fed back to the participant, researchers and the healthcare provider. Conversely, a mixture of 10 studies (both pilot and full RCTs) that were not significantly effective at improving adherence utilised at least two or more intervention components.^4^,^36^,^46^,^53^,^55^,^58^,^60^,^64–66^ Seven of these 10 were not theory-based,^36^,^46^,^58^,^60^,^64–66^ and those that were either had low intervention completion^4^,^53^ or were a pilot study with low sample size.^55^

In seven of the nine significantly effective studies, the application of technology was predominant. Two studies offered telephone-based education sessions.^48^,^50^ Reminder systems through text messages^54^ and a “Pillsy bottle” with audio-visual reminders (where the bottle blinked and beeped if unopened at the scheduled dosing time, every 10 minutes for up to an hour)^57^ were also utilised. Both disease activity monitoring^48^ and adherence monitoring^57^ only significantly impacted adherence in two studies when as the primary intervention, monitoring was combined with a technology-assisted approach. In two other studies, monitoring of disease activity^47^ and medication taking behaviour,^62^ was helpful as an additional secondary intervention. Another study using technology and counselling was tailored to participants’ needs with a view to empowering patients, yet did not significantly affect adherence.^53^ As secondary interventions, tools (such as diaries, calendars, and advice sheets) were most commonly used, with 80% of studies in this category being significantly effective. Frequent recording and checking of intended medication adherence via a calendar were successful in significantly improving medication taking.^45^ Only 37.5% of interventions with educational components resulted in significant adherence improvements. Of those which were effective, education was offered on a 1:1 basis with three to four sessions lasting between 20–40 minutes^45^,^48^,^59^ or educational information was accessible for at least 12 weeks or more.^47^,^48^

### Theoretical Basis

There were inconsistent results with a wide range of theoretically-based interventions. Only 10 interventions offered a theory to explain adherence behaviours; of which four reported significant adherence improvements post intervention. The four theories were: a) the health belief model, where perception of threats, barriers and cues predict health behaviours,^59^ b) the health action process approach, applying coping planning techniques to overcome barriers to adherence,^45^ c) CBT; emphasising learning new skills helpful in disease management^49^ and d) transtheoretical model with motivational interviewing (MI) and multiple stages facilitating behaviour change, supported by different strategies minimising resistance and maximising engagement.^62^ Each of these approaches theoretically underpinned one intervention with statistically significant effects on adherence. Two studies^64^,^66^ utilised MI as the theoretical basis of the intervention, yet neither explained the evidence-basis for its use. Both reported non-significant results.

Intervention development was explored in most depth with the PAPA theoretical framework,^4^ outlining the application of a range of recommendations, guidelines and research. Advisory panels of experts and patients were consulted, and further usability testing was carried out. Behaviour change techniques were used to develop content, context, and delivery vehicle; however, no significant effects on adherence were reported. Similarly, CBT and MI,^51^ health behaviour and social learning theory,^52^ elaboration likelihood model (ELM),^53^ systems theory^55^ and cognitive theory of multimedia learning process^63^ were all incorporated within interventions, yet led to non-significant effects on adherence. The remaining 12 (58.3%) studies did not utilise a theory to develop their intervention.^36^,^46–48^,^50^,^54^,^56–58^,^60^,^61^,^65^ Despite this, almost half (five) of these significantly improved adherence.^47^,^48^,^50^,^54^,^57^

### Completion Rates

Completion rates of both intervention and primary outcome were variable across studies, ranging from 38.9%^51^ to 100% for primary outcome completion^62^ and from 42.5%^64^ to 100%^45^,^60^,^62^ in intervention completion. Within those interventions significantly improving adherence, intervention completion rates were above 93.5% and primary outcome completion rates were above 73% for full RCTs,^45^,^47^,^59^ with unreported completion for one study.^49^ For pilot RCTs, intervention completion rates were above 81% and above 78% for primary outcome completion rates,^48^,^54^,^57^,^62^ again with one not reporting rates.^50^

For non-significant interventions, completion was 86% and above for intervention, primary outcome or both, for only five studies.^58^,^60^,^61^,^63^,^66^ Poorest completion was seen in a pilot RCT with 38.90% primary outcome completion,^51^ closely followed by 39.90%,^56^ 42.50%,^64^ and 42.60%.^4^ These studies used a variety of subjective and objective measures of adherence, although the commonality was the subjective measure, this being the Morisky Medication Adherence Scale,^68^ the Medication Adherence Report Scale^69^ or both.

## Discussion

Overall, just over a third (37.5%) of studies reviewed reported statistically significant difference in adherence and four of these were full RCTs, meaning there is an extremely limited evidence base. Of the three inflammatory conditions considered, IBD fared worst, with two of eight (25%) IBD interventions being effective in significantly improving adherence compared with controls. Two of the four (50%) MS studies (both pilot) and five of 12 (41.7%) RA studies reported statistically significant effects on adherence improvement.

This review has highlighted that a range of approaches have been applied to improve adherence. Results were contradictory in that both effective and ineffective interventions each used multiple approaches. However, the only consistent factor across all nine significantly effective studies was utilising at least one predominant intervention approach supported by at least one additional approach.^45^,^47–50^,^54^,^57^,^59^,^62^

### Tailoring

Tailored approaches were primarily used in the successful interventions. Tailoring involved adapting information to a patient’s information-processing style and learning (such as need for cognition, affect or for autonomy). Mode of delivery was tailored or the patient was encouraged to adapt the intervention to their needs.^78^ Personalised tailoring, as opposed to group support,^59^ enabled individual needs to be the central focus, increasing likelihood of helping change behaviour.^79^ These adaptive encouragement strategies applied in adherence research exert more persuasive effects,^80^,^81^ facilitating self-efficacy to address barriers.^53^ The tailored RCTs largely had good power; sample sizes of 85 and above (with the exception of two pilots), intervention completion over 73%, significance level of 0.05 and below and overall good effect size, thus being more likely to lead to positive results.

### Clinician Interaction and Training

Tailoring was found to be most effective when delivered by a trained healthcare professional, mainly nurses, applying consistent communication strategies.^53^,^65^ A high percentage of non-adherence was found to be associated with the physician–patient interaction.^48^ This is in line with previous findings that infrequent, poor communication between patient and clinician can potentially lead to 19% lower medication adherence.^14^,^82^,^83^ Patient interactions with pharmacists, for example, can be restricted and unfamiliar, impacting upon relations and subsequent adherence. All four studies/arms where pharmacists facilitated adherence support resulted in a non-significant difference,^54^,^64–66^ and in one arm was found to have a lower proportion of adherent patients compared to the control arm.^54^ To facilitate a skills-driven, disease-focused intervention, healthcare professionals require depth of knowledge of both psychological and physical demands of the disease and treatment.^52^ This is vital in assisting individuals to improve disease self-management and adherence^84^ and may in turn be a useful strategy for improving the patient–physician relationship.^85^,^86^ A high priority for research is training accessible healthcare practitioners in health decision–counselling methods and patient education skills.^49^

### Patient Education

Patient education was incorporated within several successful interventions, but was most effective when integrated with technology, monitoring and psychological behaviour change via multiple sessions or long-term accessible information. Self-management and treatment decision-making in RA resulted from an educational and counselling intervention merged with a fitness programme.^49^ Similarly, a wide range of web-based learning methods proved to be beneficial in the 12 month IBD coach intervention.^47^

The challenge of an integrated approach incorporating multiple elements is difficulty in determining cause, impact, and extent. Overcoming this, one study provided their control group with intervention features of action plans and an educational curriculum, ensuring the impact of monitoring, prompting and interaction of a home telemedicine could be assessed.^46^ Action and coping planning have also been used successfully within previous interventions to significantly impact adherence.^87^

Adherence promotion through patient education as a single approach has limited effect.^88^ Former findings^89^,^90^ and this review^45^,^55^,^56^,^61^,^63^ have shown education alone has inconsistent short-term benefits in facilitating adherence, even if patients’ knowledge about disease and treatment is improved.^54^ If a non-adherent patient already has good knowledge of their disease and treatment, specific educational interventions may be inappropriate or may skew results on adherence impact.^47^ When two methods of patient education were used over 12 weeks to target medication taking in RA participants,^60^ this led to no significant difference. Health literacy was not tested, which could indirectly impact comprehension and utilisation of study educational materials and clinical resources. If providing participants with educational resources, their understanding of and ability to use them must be ensured to promote effectiveness.^48^,^59^,^63^,^91^ Studies thus far have found that to achieve sustainable impact, educational sessions and materials should be conveniently accessible for a substantial period, either a minimum of three one-on-one sessions, each lasting at least 20 minutes,^45^,^48^,^59^ or information being available for 12 weeks or more.^47^,^48^ Even if a patient chooses not to take medication for valid reasons, such as side effects or long-term effects of medications, these reasons still must be understood to allow treatment options to be explored.

### Technology-Based Interventions

Technological and web-based interventions have become increasingly popular, with benefits demonstrated by five of the nine successful studies.^47^,^48^,^50^,^55^,^60^ This included a reduction in flare-ups, emergency visits and surgeries through systematic implementation of educational, supportive and monitoring strategies of patients and disease activity.^47^ Digital systems were accessible, feasible and modifiable,^4^ with ease of implementation.^47^,^48^ Continuity of care was established with fewer geographical restrictions, particularly in remote areas,^92^ reducing time travel to in-person clinics and related costs such as hospital parking.

Electronic diaries used as an adherence tool have been found to motivate patients in medication taking, maintaining a patient-centred focus.^50^ This may improve interactions between healthcare providers and patients.^37^,^47^,^90^ It also provides opportunities for personalised approaches to current models of care.^4^ Artificial intelligence adherence programmes can support strained health systems, minimising demands on outpatient and inpatient settings due to reduced relapses,^4^ whilst being safe and moderately cost-effective.^93^,^94^

This growing trend in online chronic disease programme management has demonstrated effectiveness through improved healthcare outcomes in a range of chronic conditions,^95^ including congestive heart failure,^96^ diabetes,^97^ chronic obstructive pulmonary disease^98^ and IBD.^47^,^48^,^99–102^Technological interventions are not always successful, however, with this being a main approach in five ineffective interventions in this review.^4^,^37^,^46^,^54^,^59^ Of course, patients who do not have internet access or are unable to use an appropriate electronic device may be excluded.^37^,^46^,^47^,^102^ One home telemanagement system^46^ required home installation, potentially impacting recruitment and attrition due to technical difficulties, questioning whether such a system would be favoured long-term. High attrition rates have also been found in intervention arms of more recent ineffective web-telemedicine studies,^4^ with the exception of one RCT,^48^ possibly due to the reminder system within the intervention and short 12 week follow-up period. Telemedicine systems may also be prone to functional errors, being based on incorrect design assumptions developed with minimal input from patients and clinicians.^103^ This can lead to inconsistent results for disease outcomes whilst being dependent on study type, design, patient population and healthcare system in which they are applied.^98^,^100^,^101^,^104^

A web-based system can also have a low impact on an individual’s behaviour as opposed to face-to-face sessions with a clinical professional or researcher,^4^ with few telemedicine systems being implemented in everyday clinical practice^47^ pre-COVID. However, restricted person-to-person contact during the pandemic led to substantial acceleration in development and implementation of digital healthcare.^105^ Systems have become more user-friendly, typically, with rising numbers of people accessing the internet.^106^ Intervention usability and acceptability is increasingly critical, with an intervention needing to be accessible and easy to use for all, including clinical staff and researchers.^48^ If participants recognise the importance of an intervention and the impact this has on their understanding and condition, it is more likely to have a positive effect on their adherence, and attrition rates. One significantly effective technology-based intervention was rated as highly successful by more than 90% of intervention participants, with no attrition for the intervention or primary outcome completion.^63^ The World Health Organization (WHO) acknowledges the necessity of digital healthcare, providing recommendations for its use,^107^ yet a framework for the development, evaluation and implementation of eHealth adherence interventions is still lacking. This would be beneficial for future technological research in adherence promotion.^14^

### Theory

Use of appropriate theory for evidence-based adherence interventions has been suggested by the UK Medical Research Council framework for developing complex interventions^108^ and the UK National Institute of Health and Care Excellence.^109^ In support of this, one significantly effective intervention^45^ was theoretically underpinned by the health action process approach and mapped to behaviour change techniques.^110^ The PAPA theoretical framework was used to design an online intervention to change IBD adherence-related medication beliefs and concerns.^4^ However, adherence results were inconsistent.^111^

The argument that theory-based programmes demonstrate more effectiveness at promoting behaviour change compared with atheoretical approaches^53^ is thus questionable, with almost half of the significantly effective interventions reviewed not being theory-based.^47^,^48^,^50^,^55^,^58^ As only 10 (41.7%) of the RCTs reviewed reported a theoretically-based intervention (five being pilot studies and five full RCTs), this scoping review reinforces how theory has typically been overlooked in intervention development and evaluation.^112^ Studies rarely examine theory-related mechanisms to explain medication adherence.^46^ Theory-based interventions require further investigation.^45^,^113^

## Limitations

There are several limitations of this review. The multiplicity of RCTs with varying designs and data across diverse clinical services, countries and continents has led to difficulties in identifying which intervention components and modes of delivery were most effective. Firstly, only three studies had inclusion criteria for low adherence at baseline,^61^,^62^,^65^ identified through pill counts and questionnaire completion. Screening for significantly poor adherers prior to recruitment is recommended practice to ensure sufficient capacity to benefit from the intervention. Monitoring any phenomenon such as “regression to the mean”^114^ and “Hawthorne effects”^115^ is equally important.

Identifying and subsequently monitoring adherence using self-report methods is typical, used by 75% of the studies, with measures such as the MARS being significantly associated with objectively assessed medication adherence.^54^ However, it is also commonly acknowledged they are more prone to memory or social desirability bias and lack objectivity.^45^ Such subjective measures may have led to participants under-reporting non-adherence,^57^ creating a ceiling effect on adherence improvements.^4^ Conversely, in another study, over-estimated adherence levels were reported,^4^ higher than previous in IBD research,^116–118^ which in part may be attributed to a lack of or non-specific operational definitions of medication adherence at intake. The inconsistency of studies measuring adherence based upon a single medication or across all medications, in all formats, adds further complexities. A more objective measure of adherence could therefore be beneficial; for example, urine levels of 5-ASA medication possession ratio,^57^ serum concentration of medication or electronic drug monitoring,^45^ also particularly useful for investigating disease activity in IBD and RA.

Cautious interpretation of results is required for several reasons. More than 50% of studies showing a significant effect on adherence were pilot studies, meaning typically low sample sizes. Some studies had low completion rates or unclear intervention completion and only five applied the intention-to-treat principle meaning their data may overestimate the true magnitude of effect. Per protocol analysis results in greater strength of association and increased biases. It is therefore recommended by the CONSORT guidelines for reporting of RCTs^119^ that both intention-to-treat and per protocol analyses should be reported, to enable readers to make their own interpretation.

Although significant adherence improvements were seen in some intervention arms, these were also evident in some control arms,^48^ suggesting change may not be due to the intervention. In addition, when no significant difference between groups was found, it is uncertain whether this is caused by attrition or ineffective treatment. This reinforces the criticality of the design, power and inclusion criteria of the study, monitoring adherence and fidelity to the intervention and adequate follow-up rates. Finally, comparison of variable intervention durations, intensities and many lacking a theory basis may also somewhat limit applicability and relevance of results.

## Conclusion

Improved medication adherence has been found to enhance long-term inflammatory disease outcomes, promoting health, including quality of life. There have been a range of interventions aiming to boost adherence in the inflammatory conditions of IBD, MS and RA, yet the vast majority have been ineffective. Adherence support interventions in inflammatory conditions therefore need improving.

Intervention development would benefit considerably from healthcare professionals trained in adherence support. Their role in helping to promote in-depth understanding of inflammatory conditions and associated medications, whilst offering consistent and/or long-term patient-based, interactive approaches targeting a patient’s personalised needs, is essential. This has potential to simultaneously enhance the patient–clinician relationship, facilitating open, honest discussion and improved medication adherence.

The application of theory in medication intervention development has been extremely limited, and when used, demonstrates mixed evidence. Theoretically-based interventions therefore require further exploration so the impact can be more accurately assessed. Increasingly popular technology-based routes of intervention delivery must be accessible, user-friendly, practical and functional, which can be synchronised with useful self-management supportive tools offering patient feedback. Potential interventions ideally will be efficient yet cost-effective and evaluated in adequately powered RCTs, with the optimal goal to truly benefit patients, professionals, and services alike.

This article was a poster presentation at the 18th Congress of European Crohn’s and Colitis Organisation, Copenhagen, Denmark, 2023.

## Abbreviations

bDMARD, biological disease modifying antirheumatic drugs; CD, Crohn’s disease; CQR-19, Compliance Questionnaire Rheumatology; DMT, disease modifying therapy; ELM, elaboration likelihood model; EMF, electronic monitoring feedback; HAT, home automated telemanagement; IBD, inflammatory bowel disease; ITT, intention-to-treat; MAQ, Medication Adherence Questionnaire; MARS, Medication Adherence Report Scale; MEMS, medication electronic monitoring system; MI, motivational interviewing; MMAS, Morisky Medication Adherence Scale; MPR, medication possession ratio; MS, multiple sclerosis; MSP, (tele) Medicine Patient Support Programme; MTX, methotrexate; MUSP, Musculoskeletal Ultrasound Programme; PAPA, perceptions and practicalities approach; PROMs, patient-reported outcome measures; PC, pharmacist counselling; RA, rheumatoid arthritis; TAU, treatment as usual; TM, text message; TMI, tailored multimedia intervention; UK, United Kingdom; USA, United States of America; VAS, visual analogue scale.

## Data Sharing and Availability

The authors confirm that this manuscript, in addition to the findings, tables, and figures, have not been published previously elsewhere or are under consideration for publication. The data underlying this article are available in the article and in its online Supplementary Material.
